# Research into the mechanism of intervention of SanQi in endometriosis based on network pharmacology and molecular docking technology

**DOI:** 10.1097/MD.0000000000030021

**Published:** 2022-09-16

**Authors:** Zhiheng Lin, Weisen Fan, Xiao Yu, Jinxing Liu, Pengfei Liu

**Affiliations:** a Shandong University of Traditional Chinese Medicine, Jinan, Shandong, China; b Affiliated Hospital of Shandong University of Traditional Chinese Medicine, Jinan, Shandong, China.

**Keywords:** endometriosis, molecular docking technology, network pharmacology, Sanqi

## Abstract

By using network pharmacology and molecular docking technology, we have explored the mechanism of action of Sanqi in the treatment of endometriosis (EMS), in order to provide reference for clinical studies of Chinese medicine treatment of Ems and Chinese medicine pharmacology.

**Methods:**

There are 123 intersecting targets between the active ingredients of Sanqi and disease targets. In the Protein-Protein Interaction network, Jun proto-oncogene, AP-1 transcription factor subunit, tumor necrosis factor, interleukin 6, etc., are the core proteins. The top 20 genes ranked by degree have been analyzed according to the Kyoto Encyclopedia of Genes and Genomes pathway and Gene Ontology analysis, and 20 pathways have been identified.

**Results:**

On the Kyoto Encyclopedia of Genes and Genomes pathway, the most important part is the phosphatidylinositol 3’-kinase-Akt signaling pathway, and on the Gene Ontology pathway, it is the Heme binding. The top 3 targets docked to quercetin have a certain affinity when it is docked to their degree value. Among the chemical components of Sanqi, quercetin has the most targets, suggesting that it may play a major role in the treatment of EMS.

**Conclusion:**

The results of molecular docking provide further evidence of the potential role of Sanqi for EMS. Overall, our study provides a new direction for the treatment of EMS and provides the basis for Sanqi as a drug for the treatment of EMS.

## 1. Introduction

Endometriosis (EMS) is the most common problem in women of child-bearing age.^[1]^ The cause is the overgrowth of endometrial glands and the stroma in areas other than the lining of the uterus.^[2]^ The clinical manifestations include pelvic pain, an irregular menstrual period, and an abnormal menstrual flow, which can result in infertility and mental illness, as well as malignant cancers.^[3]^ It is easy to recur and causes a greater economic burden on patients.^[4]^ At the present time, EMS is a major public health concern of global concern.^[5]^

In terms of treating EMS, traditional Chinese medicine offers many advantages, such as reducing its recurrence rate, reducing pain, and having fewer side effects.^[6,7]^ EMS belongs to the category of Zheng Jia (blood stasis) diseases according to traditional Chinese medicine, where its disease mechanism has been described as “blood stasis” for a long period of time.^[8]^ Traditional Chinese medicine is usually used by Chinese doctors in order to remove “blood stasis” as part of their treatment.^[9]^ Sanqi is referred to as a “Pharmacy.”^[10]^ “BenCaoGangMu” records^[11]^ that Sanqi has the effect of reducing “blood stasis” and hemostasis, and it can be used for the treatment of traumatic bleeding, traumatic injury, and “ZhengJia” Class diseases. Pharmacological research indicates that^[12,13]^ there are various active ingredients in Sanqi, such as panax notoginseng saponins, panax notoginseng elements, which may have hemostasis, anti-inflammatory, effects, etc. In this research, we utilized the research method of network pharmacology to predict the mechanism of action of Sanqi on EMS, with the hope of providing reference for further experimental studies.

## 2. Resources and Methods

### 2.1. Sanqi Chemical Composition Retrieval

In order to clarify the chemical components contained in Sanqi, we searched the Traditional Chinese Medicine Systems Pharmacology database^[14]^ (http://tcmspw.com/tcmsp.php, updated May 31, 2014) for all the active ingredients contained in Sanqi, and based on the active ingredients in the database, we queried the target. And we further searched for important targets related to “Sanqi” on target prediction websites such as SwissTargetPrediction (http://swisstargetprediction.ch/), DrugBank (https://go.drugbank.com/), and PubChem (https://pubchem.ncbi.nlm.nih.gov/). A database such as this will facilitate our research into the modern pharmacology of Chinese medicine and assist us in discovering the essence of Chinese medicine in treating disease. As the active ingredients of Sanqi cannot be fully studied, as well as the clear mechanism of related diseases, this study is limited to all of the chemical components of Sanqi. The study protocols were performed according to Declaration of Helsinki. The data for this study were all obtained from public databases and therefore did not require ethics committee approval.

### 2.2. Prediction of known therapeutic targets of EMS

The therapeutic targets of EMS were collected from 3 sources. Among these are GeneCard (https://www.genecards.org/), DisGeNET (https://www.disgenet.org/), and OMIM (https://omim.org/). Once the EMS target has been retrieved, we can take the union retrieved from the 3 databases. GeneCard database,^[15]^ DisGeNET database,^[16]^ and OMIM database^[17]^ offer comprehensive, free, and authoritative records of genes and are widely used in network pharmacology research.

### 2.3. Target protein and target gene conversion

A set of target points derived from the Traditional Chinese Medicine Systems Pharmacology database is entered into the UniProt database (https://www.uniprot.org/), and human target genes are selected during conversion.

### 2.4. Construction of the intersection gene and protein network

We used the target genes acted upon by the EMS gene and the active ingredients in Sanqi to draw the intersection map of the two and to identify the intersection gene by the software Venny 2.1. We entered intersection genes into the String database^[18]^ to create a Protein-Protein Interaction (PPI) network, imported the PPI network into Cystoscap 3.6.0 software, and used the Cellular Angular plug-in cytoHubba to filter PPI-based central genes to draw network diagrams based on the top 20 rankings.

### 2.5. Enrichment analysis of the Kyoto Encyclopedia of Genes and Genomes pathway and Gene Ontology pathway

David Database^[19]^ facilitates the progression from data collection and analysis to biological significance and enriches target genes according to their functions. For Kyoto Encyclopedia of Genes and Genomes (KEGG) and Gene Ontology (GO) pathway enrichment analysis, enter the intersection target into the David database (https://david.abcc.ncifcrf.gov/) and use R programming language to generate a degree value bubble chart based on the top 20 targets. The higher the value of degree,^[20]^ the more critical it demonstrates that the target protein is. Use the KEGG pathway analysis website (https://www.kegg.jp/) to draw a pathway map showing the top 20 targets with the highest degree values.

### 2.6. Chemical composition-target gene, KEGG pathway-target gene interaction network diagram

Genes corresponding to 17 chemical components associated with EMS in Sanqi and the genes corresponding to the top 10 pathways analyzed by the David website were entered into Cytoscape, and a network of target genes corresponding to the chemical components of Sanqi was constructed.

### 2.7. Docking of chemical components with target protein

A chemical composition is selected for molecular docking with a target gene based on the interaction network between the chemical compositions and the target gene and the degree value. Chemical composition of Panax notoginseng was derived from PubChem database^[21]^ (https://pubchem.ncbi.nlm.nih.gov/) query, and molecular energy was minimized through Chem3D software. The target protein is downloaded from the protein data bank database (https://www1.rcsb.org/) using PyMOL software.^[22]^ The protein ligand is deleted and dehydrated. The protein molecule is hydrogenated, and finally, PyMOL software is used for visualization and docking.

## 3. Results

### 3.1. Sanqi and EMS target data

**Figure 1.**
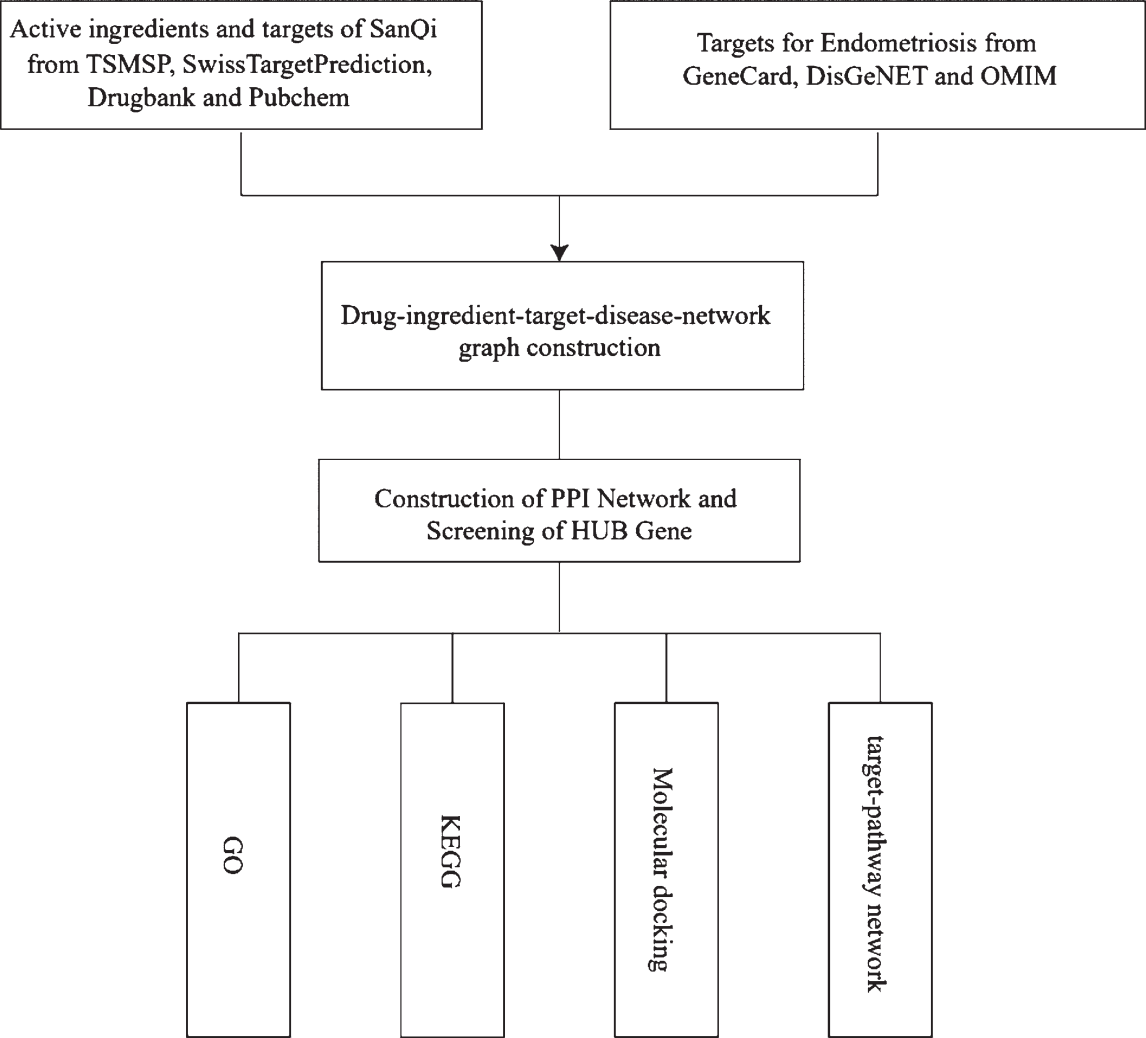
The flow chart of records search and selection. GO = Gene Ontology, KEGG = Kyoto Encyclopedia of Genes and Genomes, PPI = Protein-Protein Interaction.

**Figure 2.**
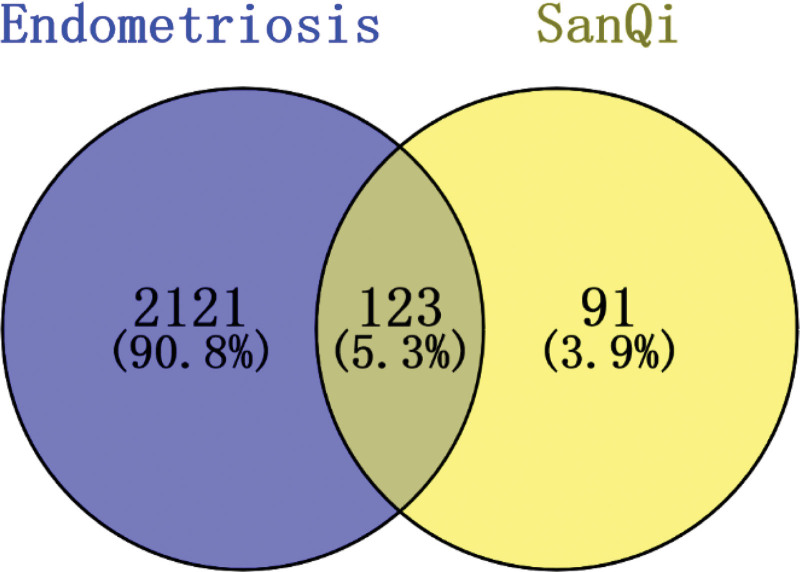
Intersection analysis of the disease targets of the Venn diagram on endometriosis and the action targets of the active ingredients of the traditional Chinese medicine Sanqi.

The Figure 1 shows records search and selection. Sanqi contains 21 active ingredients, 17 of which are associated with EMS in this research. Table 1 includes further details. UniProt has converted 214 human targets. The 3 databases produced 2244 disease targets in total. EMS and Sanqi have 123 intersection targets according to the Venny software. These details are illustrated in Figure 2. And the records sources and summarize, see Table 2. All associated references to each active ingredient see Supplementary List, http://links.lww.com/MD/H15.

**Table 1:** Active ingredient list of Sanqi.

MOL ID	Molecule name	OB (%)	DL
MOL001494	Mandenol	42	0.19
MOL001792	DFV	32.76	0.18
MOL002153	1H-Cycloprop(e)azulen-7-ol, decahydro-1,1,7-trimethyl-4-methylene-, (1aR-(1aalpha,4aalpha,7beta,7abeta,7balpha))-	82.33	0.12
MOL002879	Diop	43.59	0.39
MOL000358	Beta-sitosterol	36.91	0.75
MOL000449	Stigmasterol	43.83	0.76
MOL005344	Ginsenoside Rh2	36.32	0.56
MOL000612	(-)-alpha-cedrene	55.56	0.1
MOL000066	Alloaromadendrene	53.46	0.1
MOL000675	Oleic acid	33.13	0.14
MOL007472	(9Z,12E)-octadeca-9,12-dienoic acid methyl ester	41.93	0.17
MOL007486	ZINC01532096	50.74	0.15
MOL007498	10Z,13Z-nonadecadienoic acid	40.98	0.17
MOL007501	Panaxydol	61.67	0.13
MOL007508	α-cyperene	51.1	0.11
MOL000935	Heptanal	53.83	0.1
MOL000098	Quercetin	46.43	0.28

OB = oral bioavailability, DL = drug-likeness, MOL ID = ID information for drug components defined in the TCMSP.

**Table 2:** Search strategy for records sources and summarize.

Drug/disease	Targets sources	Number of targets
SanQi	TSMSP (https://old.tcmsp-e.com/tcmsp.php); SwissTargetPrediction (http://swisstargetprediction.ch/); DrugBank (https://go.drugbank.com/); PubChem (https://pubchem.ncbi.nlm.nih.gov/)	214
Endometriosis	GeneCard (https://www.genecards.org/); DisGeNET (https://www.disgenet.org/); OMIM (https://omim.org/)	2244

### 3.2. Protein-Protein Interaction network

**Figure 3.**
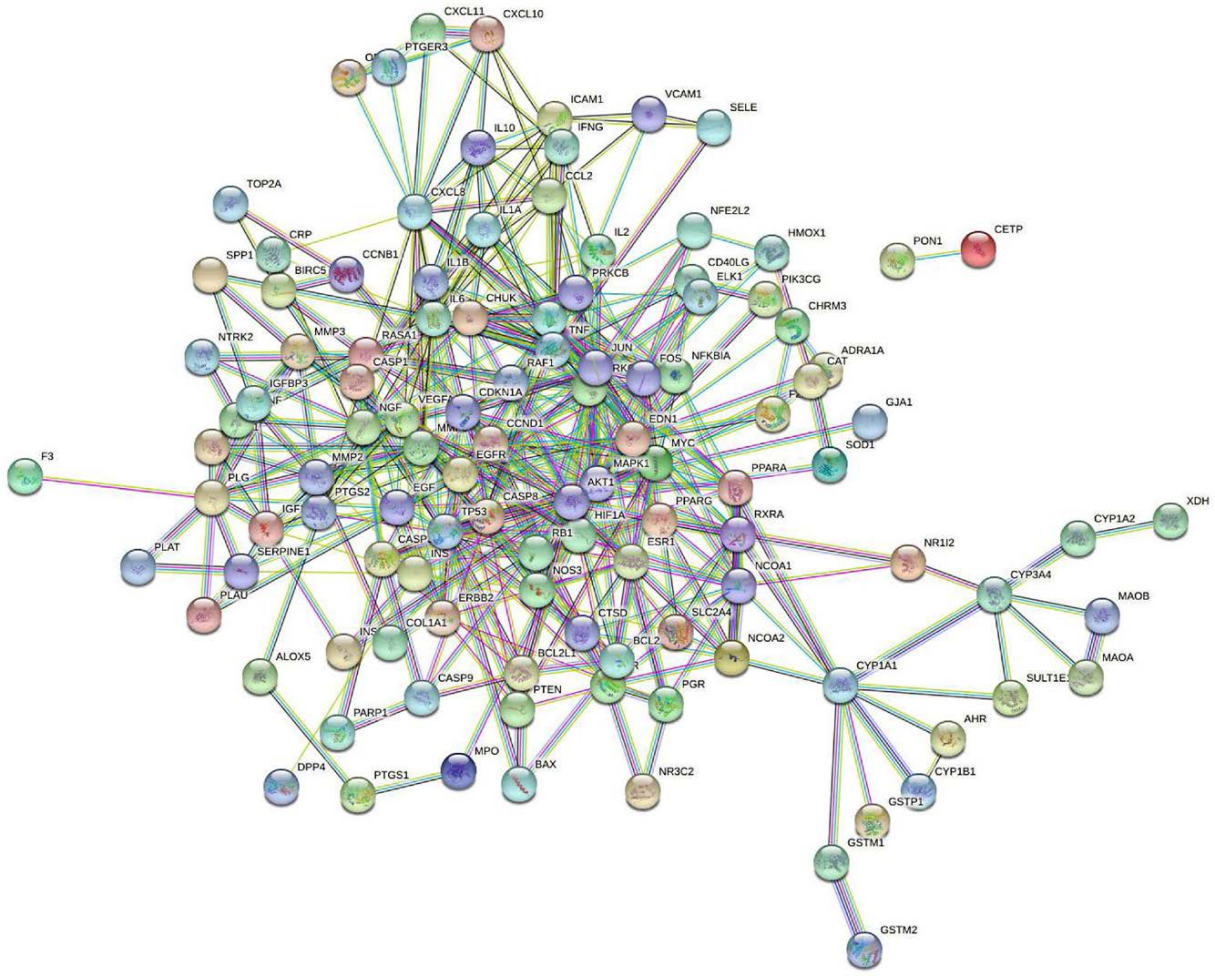
The Protein-Protein Interaction network diagram of the intersection target of endometriosis and the traditional Chinese medicine Sanqi.

**Figure 4.**
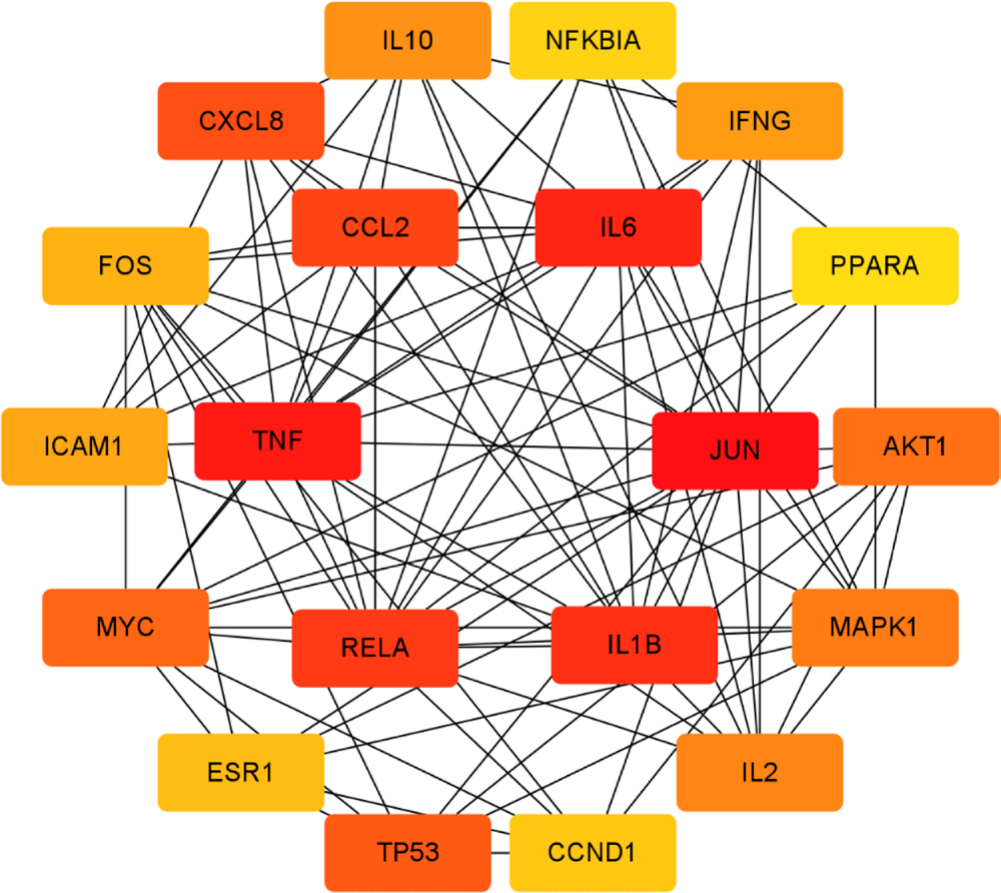
The interaction of pivot genes in the network, according to the degree value, the redder the color.

In order to make a network diagram, select “Homo sapiens” as the biological species, choose “highest confidence” (>0.9) as the minimum interaction threshold, and leave the remaining settings as default. Make a PPI network to obtain the intersection target of Sanqi and EMS PPI network diagram (Fig. 3). Number of nodes: 123, number of edges: 452, average node degree: 7.35, and PPI enrichment *P* value: <1.0 × 10^–16^. A number of genes have been identified as pivotal genes, including Jun proto-oncogene, AP-1 transcription factor subunit, tumor necrosis factor, interleukin 6, etc. Darker the color of the hub gene network graph, the more critical the gene (Fig. 4).

### 3.3. Pathway enrichment and path flow chart

**Figure 5.**
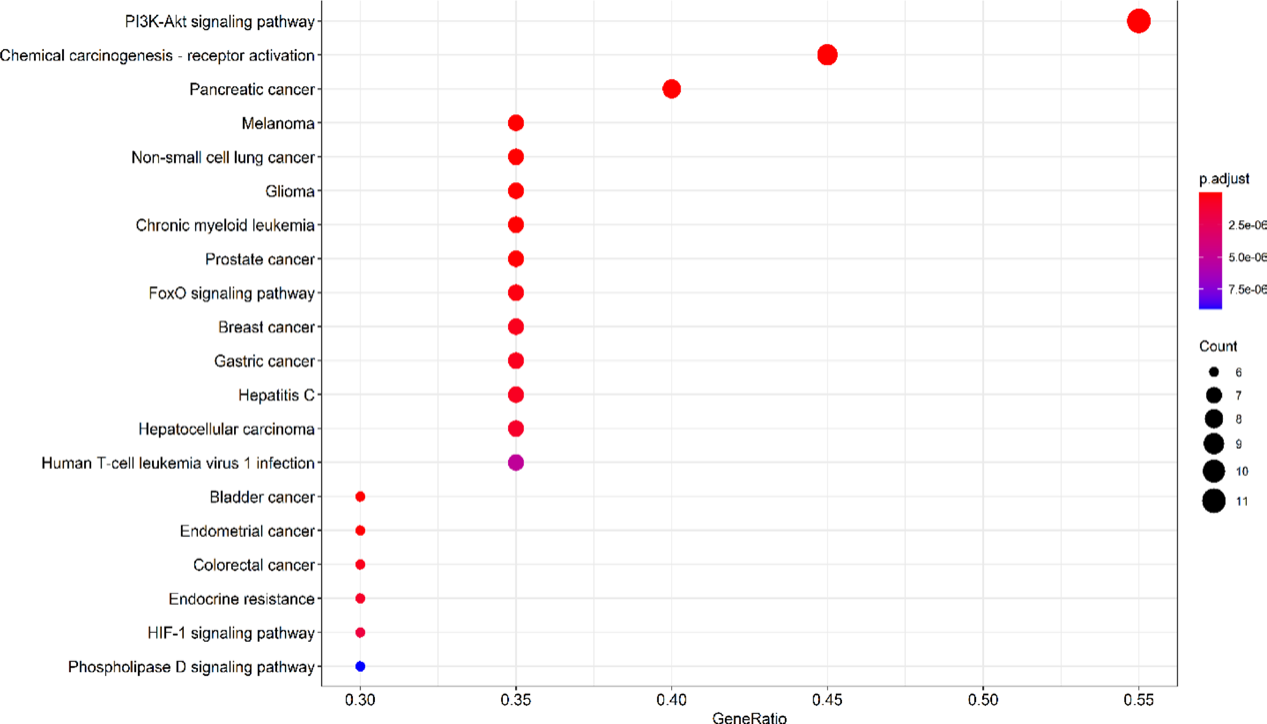
Bubble chart of KEGG pathway enrichment analysis of the top 20 target genes ranked by degree value. FoxO = forkhead box, sub-group O, HIF-1 = hypoxia-inducible factor 1, KEGG = Kyoto Encyclopedia of Genes and Genomes, PI3K-Akt = phosphatidylinositol 3’-kinase-Akt.

**Figure 6.**
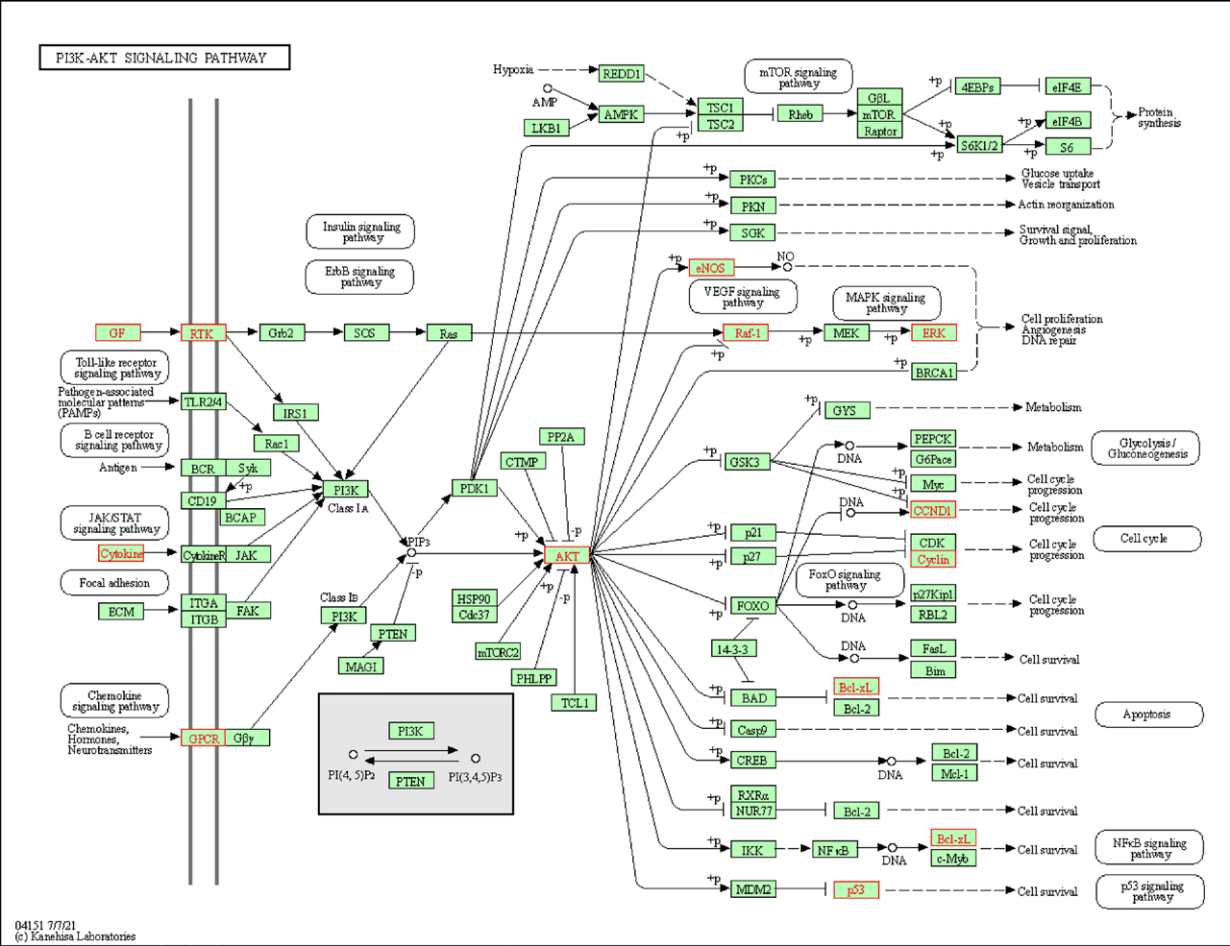
The KEGG pathway diagram of Sanqi acting on the PI3K-AKT signaling pathway. The red mark represents the target of Sanqi acting on the pathway, and the green represents other targets in the pathway. KEGG = Kyoto Encyclopedia of Genes and Genomes, PI3K-Akt = phosphatidylinositol 3’-kinase-Akt.

**Figure 7.**
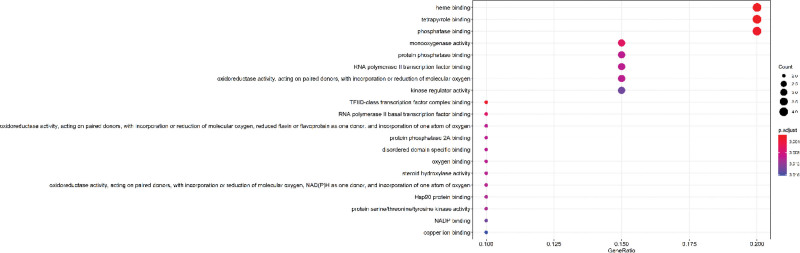
Bubble chart of GO pathway enrichment analysis of the top 20 genes of degree value. GO = Gene Ontology, Hsp90 = heat-shock protein 90, NAD(P)H = dihydronicotinamide adenine dinucleotide phosphate, NADH = dihydronicotinamide adenine dinucleotide, TFIID = The general transcription factor IID.

The top 20 degrees ranked by the KEGG pathway analysis of the phosphatidylinositol 3’-kinase-Akt (PI3K-AKT) signaling pathway illustrated in Figure 5 are the pathways with the greatest number of enriched genes and the greatest correlation. Figure 6 illustrates the PI3K-AKT signaling pathway in detail. As the number of enriched genes increases, so does the size of the bubble; the redder the color, the smaller the *P* value; and the horizontal position of the bubble represents the proportion of genes. The GO pathway in Figure 7 illustrates that the red and largest pathway diagrams correspond to the Heme binding pathway, the tetrapyrrole pathway, and the phosphate synthase pathway.

### 3.4. Construction of the composite target network

**Figure 8.**
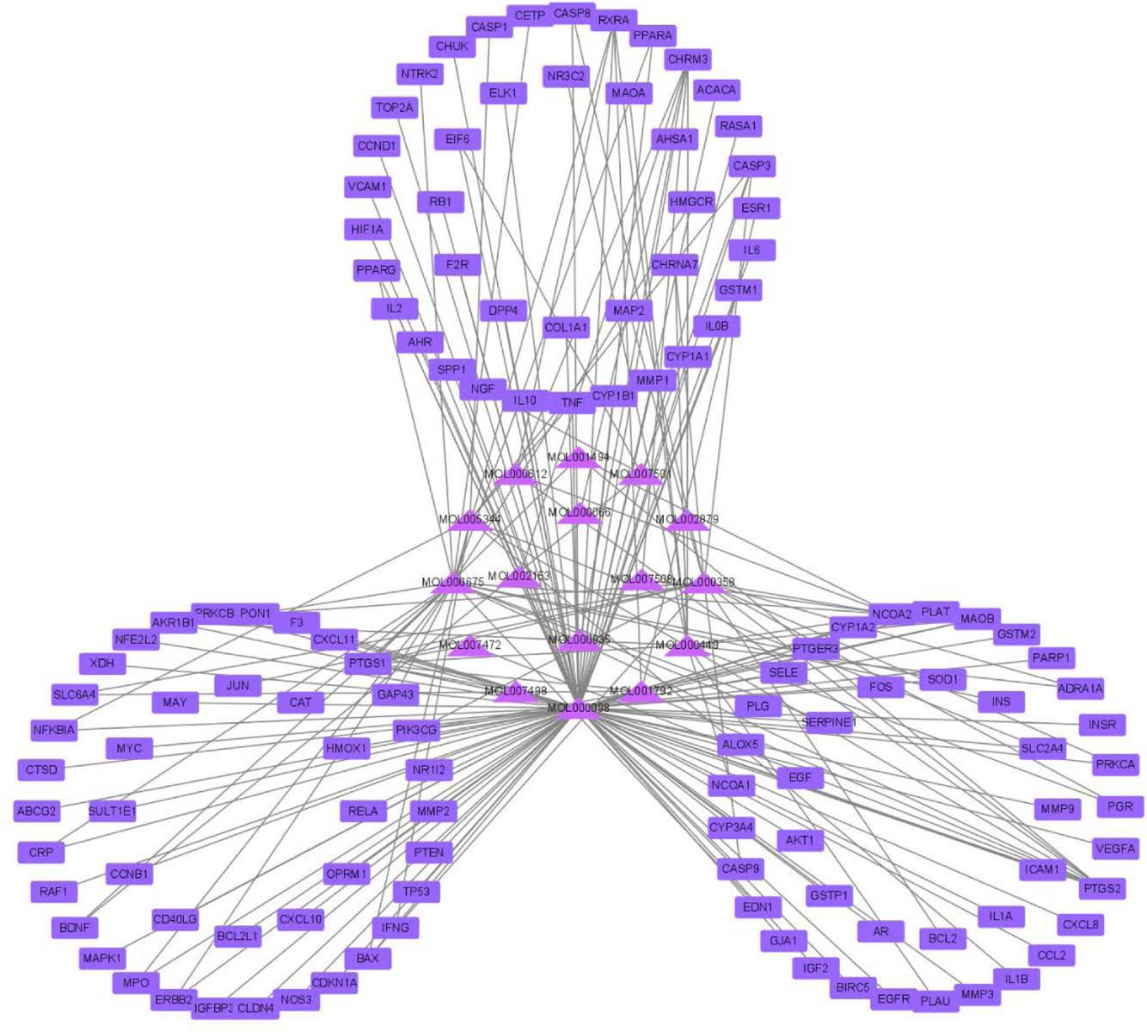
The network diagram of the multi-chemical components and multitargets of Sanqi. Purple represents the active ingredients of Sanqi, and blue-purple represents the target.

**Figure 9.**
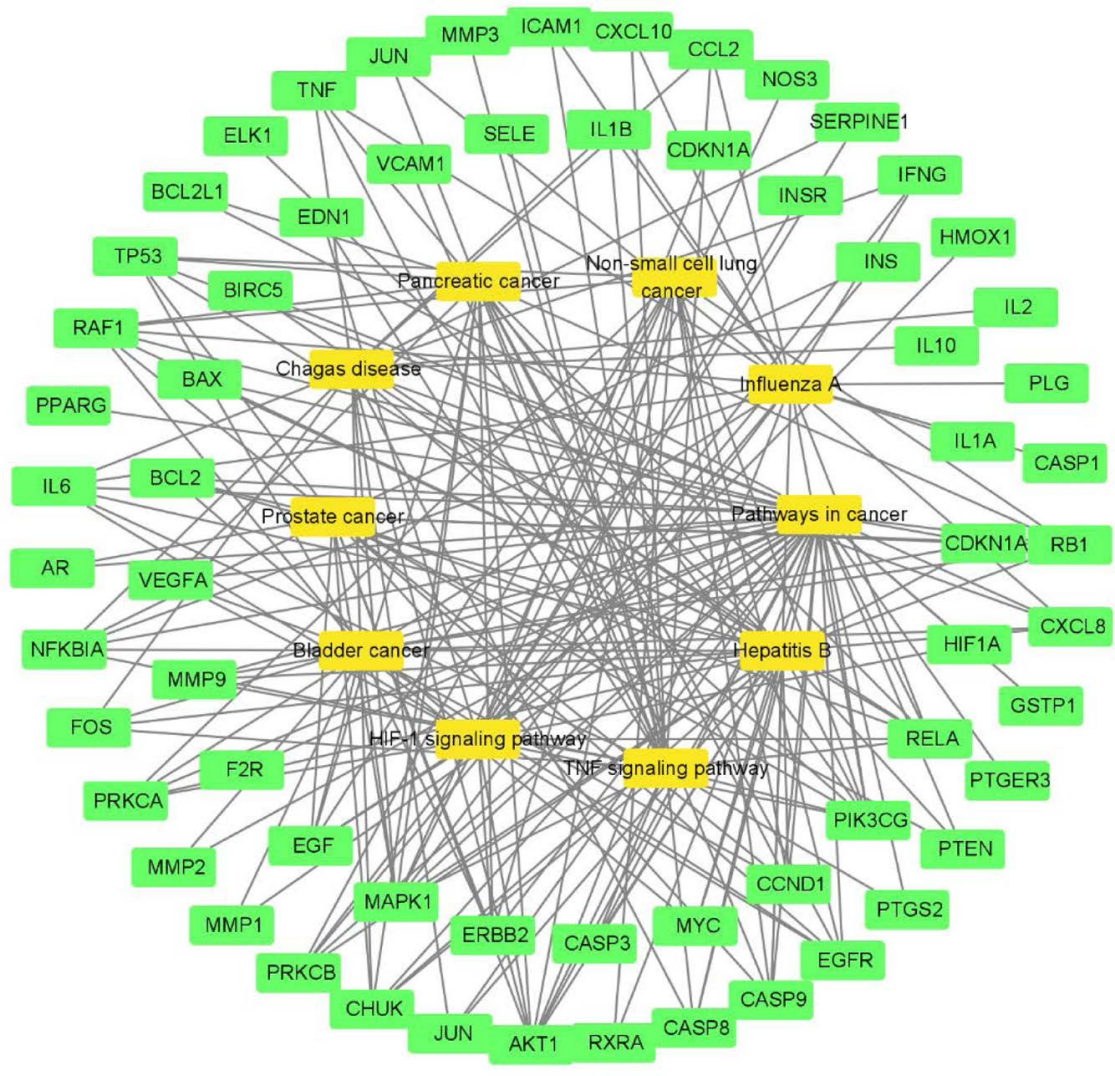
Signal pathway and target network diagram, yellow represents the KEGG signal pathway, and green represents the target enriched in the pathway. KEGG = Kyoto Encyclopedia of Genes and Genomes.

As shown in Figure 8, the multicomponent multitarget network diagram shows that the number of genes such as quercetin, oleic acid, and beta-sitosterol are relatively large. Sanqi contains a number of active ingredients, including prostaglandin-endoperoxide synthase 2 (PTGS2) and cholinergic receptor muscarinic 3. According to the multichannel multitarget network diagram in Figure 9, the top 10 KEGG pathways are enriched in mitogen-activated protein kinase 1 (MAPK1), consisting of a total of 10 pathways, phosphatidylinositol-4,5-bisphosphate 3-kinase catalytic subunit gamma, consisting of 9 pathways, and RELA proto-oncogene, NF-kB subunit (RELA), consisting of 8 pathways.

### 3.5. Molecular docking simulation

**Figure 10.**
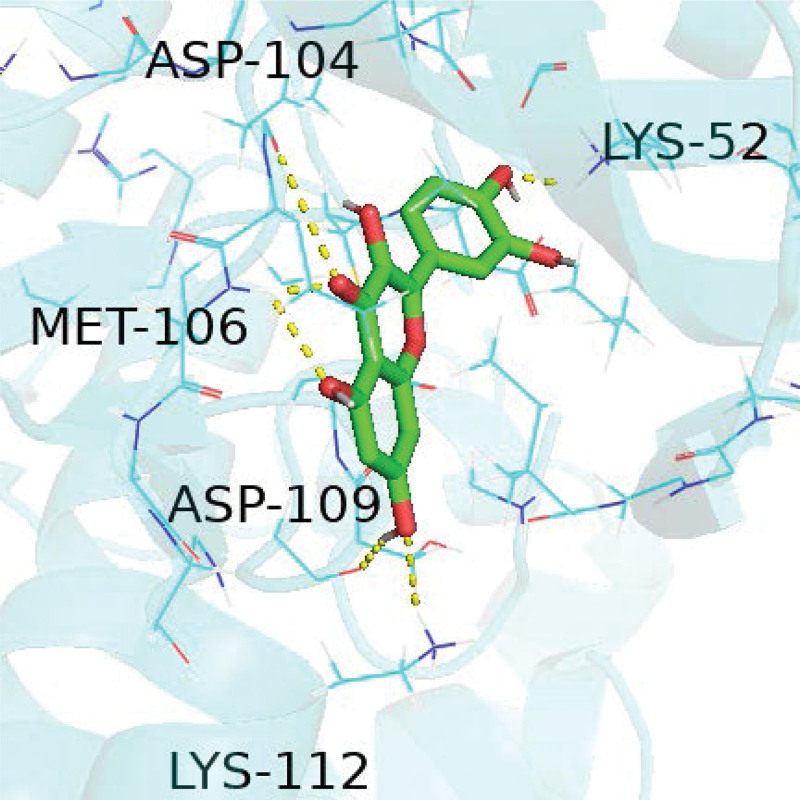
Quercetin and MAPK1 docking diagram. MAPK1 = mitogen-activated protein kinase 1.

**Figure 11.**
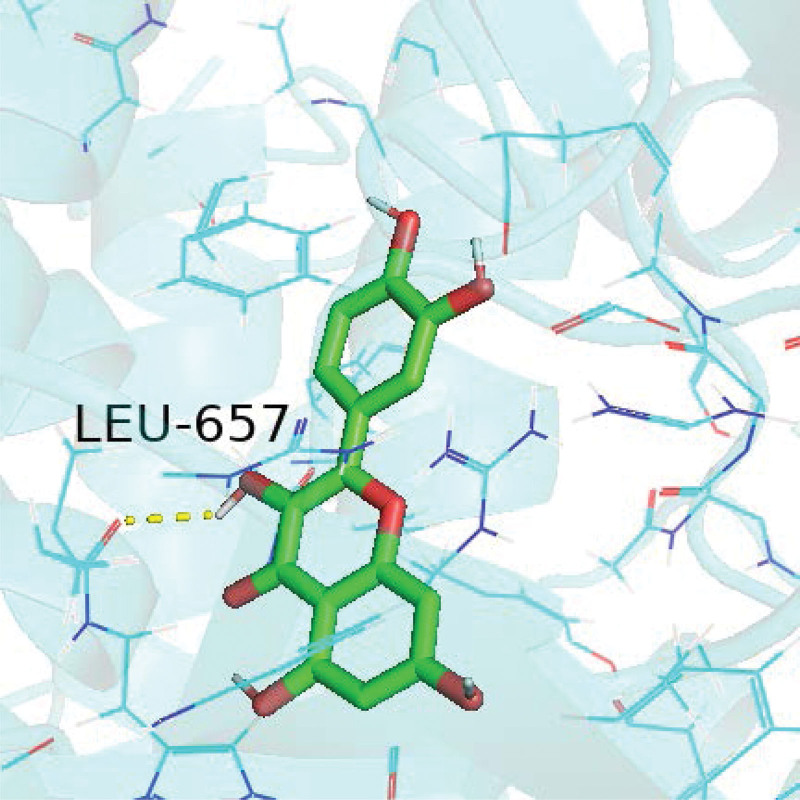
Quercetin and PIK3CG docking diagram. PIK3CG = phosphatidylinositol-4,5-bisphosphate 3-kinase catalytic subunit gamma.

**Figure 12.**
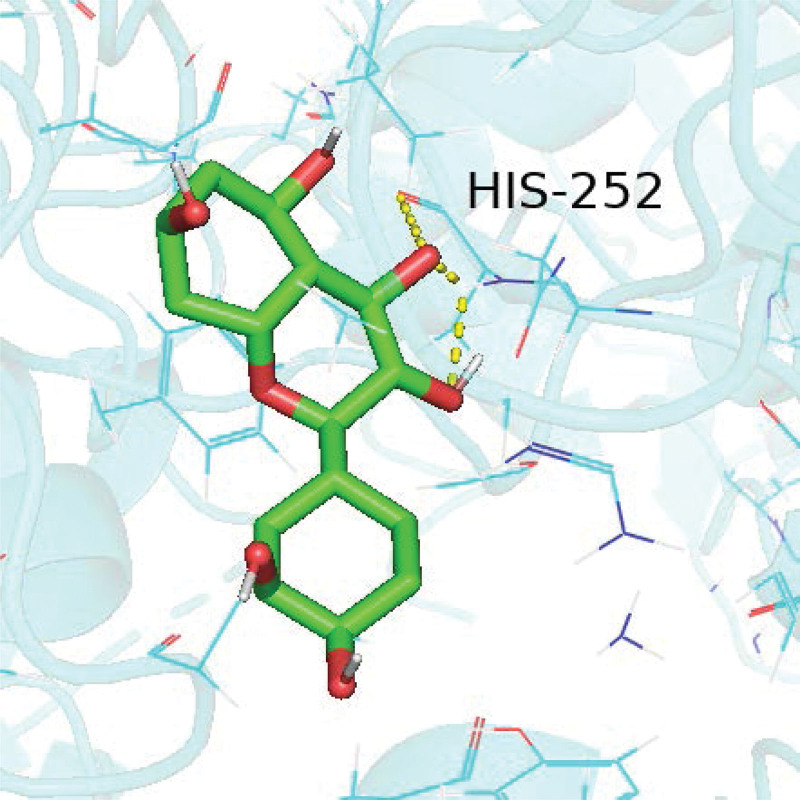
Quercetin and RELA docking diagram. RELA = RELA proto-oncogene, NF-kB subunit.

We selected the 3 targets MAPK1, phosphatidylinositol-4,5-bisphosphate 3-kinase catalytic subunit gamma, and RELA, which are most enriched in the top 10 pathways, for docking simulations with quercetin. A hydrogen bond represents the mimic action site of quercetin when it interferes with a target protein. Details are shown in Figures 10–12.

## 4. Discussion

It has become increasingly common today for traditional Chinese medicine to be used as a treatment for various diseases in other countries.^[23]^ The treatment ideas and methods of traditional Chinese medicine have, similarly to this epidemic, played a significant role in the development of disease prevention and control.^[24]^ Many factors come into play when treating diseases with traditional Chinese medicine compound prescriptions. It has been found that the interplay between chemical components, oral absorption, and in vivo metabolism of the respective drugs makes it difficult to conduct clear research on the treatment of diseases with traditional Chinese medicine.^[25]^ Accordingly, this network pharmacology study selected the meridian of traditional Chinese medicine, and the efficacy of traditional Chinese medicine is in accordance with the treatment of EMS.

According to the multicomponent and multitarget network diagrams, the 3 components of quercetin, oleic acid, and beta-sitosterol have the greatest number of targets. Several high degree targets can be affected by quercetin, such as C-C motif chemokine ligand 2, interleukin 6, tumor necrosis factor, RELA, etc. Quercetin itself has anti-inflammatory and anti-tumor properties.^[26]^ As a result, it inhibits tumor growth by increasing autophagy and apoptosis and inhibiting angiogenesis.^[27]^ Ectopic endometrial tissue of EMS has characteristics similar to those of tumor tissue, including invasiveness, adhesion, and metastasis.^[28]^ Animal experiments have found that quercetin reduces Estrogen levels in mice^[29]^ suffering from EMS when it is used to treat the condition. EMS is estrogen-dependent and proliferates continuously in response to estrogen.^[30]^ Consequently, quercetin may have a therapeutic effect on EMS. In recent years, research on the anti-tumor properties of beta-sitosterol has been extensive. Beta-sitosterol has analgesic, anti-inflammatory, and anti-tumor properties.^[31]^ However, in terms of interventions for EMS, research on the effect of oleic acid and beta-sitosterol on EMS is inconclusive. This is because both of these components can act on the target PTGS2 gene, and the reduction^[32]^ in PTGS2 expression in the cumulus cells of infertile patients caused by EMS may result in a decrease in cyclooxygenase levels, which in turn will result in impaired oocyte capacity. It can serve as a reference for further study.

The pathway that is most relevant to the top 20 targets with the highest degree is the PI3K-Akt signaling pathway, which is involved in the process of supporting the progesterone resistance effect in EMS. The pathway is also an important pathway for tumor intervention.^[33]^ As shown in Figure 6, MAPK1, AKT serine/threonine kinase 1, cyclin D1, and tumor protein p53 (TP53) are high degree targets of this pathway. As shown in Figure 6, AKT is the core target of the PI3K-AKT signaling pathway, and its up-regulation may be associated with EMS pathogenesis.^[34]^ It will act on the important tumor gene MDM2 proto-oncogene, and MDM2 proto-oncogene will affect the TP53 gene transcription protein P53 via the P53 signaling pathway.^[35,36]^ According to the research,^[37]^ the positive rate of P53 protein produced by TP53 gene in normal human endometrial tissue is higher than that in abnormal endometrial tissue from Ovarian endometriosis cyst patients. It has been shown that the P53 protein induces apoptosis,^[38]^ indicating that the abnormal expression of this gene is closely related to the abnormal hyperplasia of EMS. Quercetin can act on AKT serine/threonine kinase 1. Quercetin’s ability to influence EMS via the above pathways remains to be investigated. In addition, the results of molecular docking provide further evidence of the potential role of SanQi for EMS.

## 5. Conclusion

In summary, network pharmacology shows that the main active ingredients of Sanqi, especially quercetin, oleic acid, and beta-sitosterol, can interfere with multiple targets. Pancreatic cancer, PI3K-Akt signaling pathway, non-small cell lung cancer, and other pathways play a role. Molecular docking shows that quercetin can interfere with 3 high degree targets, which indicates that quercetin may play a major role in the treatment of EMS by Sanqi.

## Author contributions

ZL and WF conceived and designed the study; ZL and XY searched the related articles; ZL, WF, XY, and JL analyzed the data; ZL, XY, and JL wrote the manuscript. PL supervised the whole process. All authors read and approved the final manuscript.

## Supplementary Material


