# Supplementary material for: Research into the mechanism of intervention of SanQi in endometriosis based on network pharmacology and molecular docking technology
Source: Medicine (Baltimore). 2022 Sep 16;101(37):e30021. doi: 10.1097/MD.0000000000030021 (PMC9478308; doi:10.1097/MD.0000000000030021)
Supplement: Supplementary file 1 [file medi-101-e30021-s001.pdf]

Associated references to each active ingredient list of Sanqi

Molecule name	References
Mandenol	Huo M, Ma L, Liu G. Exploring the mechanism of Yixinyin for myocardial infarction by weighted co-expression network and molecular docking [published correction appears in Sci Rep. 2021 Dec 21;11(1):24459]. Sci Rep. 2021;11(1):22567. Published 2021 Nov 19. doi:10.1038/s41598-021-01691-8
DFV	Chen M, Sun Q. Systemic pharmacology understanding of the key mechanism of Sedum sarmentosum Bunge in treating hepatitis. Naunyn Schmiedebergs Arch Pharmacol. 2021;394(2):421-430. doi:10.1007/s00210-020-01952-9
1H-Cycloprop(e)azulen-7-ol, decahydro-1,1,7-trimethyl-4-methylene-, (1aR-(1aalpha,4aalpha,7beta,7abeta,7balpha))-	Madhumitha G, Rajakumar G, Roopan SM, et al. Acaricidal, insecticidal, and larvicidal efficacy of fruit peel aqueous extract of Annona squamosa and its compounds against blood-feeding parasites. Parasitol Res. 2012;111(5):2189-2199. doi:10.1007/s00436-011-2671-2
Diop	Zhou C, Zhou H, Zhang F, Hao L, Guo J. Active Ingredients and Potential Mechanisms of the Gan Jiang-Huang Qin-Huang Lian-Ren Shen Decoction against Ulcerative Colitis: A Network Pharmacology and Molecular Docking-Based Study. Evid Based Complement Alternat Med. 2021;2021:1925718. Published 2021 Sep 8. doi:10.1155/2021/1925718
beta-sitosterol	He D, Huang JH, Zhang ZY, et al. A Network Pharmacology-Based Strategy For Predicting Active Ingredients And Potential Targets Of

	LiuWei DiHuang Pill In Treating Type 2 Diabetes Mellitus [published correction appears in Drug Des Devel Ther. 2020 Mar 02;14:949]. Drug Des Devel Ther. 2019;13:3989-4005. Published 2019 Nov 28. doi:10.2147/DDDT.S216644
Stigmasterol	He D, Huang JH, Zhang ZY, et al. A Network Pharmacology-Based Strategy For Predicting Active Ingredients And Potential Targets Of LiuWei DiHuang Pill In Treating Type 2 Diabetes Mellitus [published correction appears in Drug Des Devel Ther. 2020 Mar 02;14:949]. Drug Des Devel Ther. 2019;13:3989-4005. Published 2019 Nov 28. doi:10.2147/DDDT.S216644
ginsenoside rh2	Li H, Huang N, Zhu W, et al. Modulation the crosstalk between tumor-associated macrophages and non-small cell lung cancer to inhibit tumor migration and invasion by ginsenoside Rh2. BMC Cancer. 2018;18(1):579. Published 2018 May 22. doi:10.1186/s12885-018-4299-4
(-)-alpha-cedrene	Lu J, Wang H, Huang J, et al. Sesquiterpene acids from Shellac and their bioactivities evaluation. Fitoterapia. 2014;97:64-70. doi:10.1016/j.fitote.2014.05.014
alloaromadedrene	Jesionek A, Poblocka-Olech L, Zabiegala B, Bucinski A, Krauze-Baranowska M, Luczkiewicz M. Validated HPTLC method for determination of ledol and alloaromadendrene in the essential oil fractions of Rhododendron tomentosum plants and in vitro cultures and bioautography for their activity screening. J Chromatogr B Analyt

	Technol Biomed Life Sci. 2018;1086:63-72. doi:10.1016/j.jchromb.2018.04.006
oleic acid	Yang Y, Chen J, Gao Q, Shan X, Wang J, Lv Z. Study on the attenuated effect of Ginkgolide B on ferroptosis in high fat diet induced nonalcoholic fatty liver disease. Toxicology. 2020;445:152599. doi:10.1016/j.tox.2020.152599
(9Z,12E)-octadeca-9,12-dienoic acid methyl ester	Wickens DG, Davies MJ, Fairbank J, Tay SK, Slater TF, Dormandy TL. Studies on cervical intraepithelial neoplasia: the level of octadeca-9,11-dienoic acid and measurement of free radical content by electron spin resonance spectroscopy. Am J Obstet Gynecol. 1990;162(3):854-858. doi:10.1016/0002-9378(90)91023-6
ZINC01532096	Choi S, Liu X, Pan Z. Zinc deficiency and cellular oxidative stress: prognostic implications in cardiovascular diseases. Acta Pharmacol Sin. 2018;39(7):1120-1132. doi:10.1038/aps.2018.25
10Z,13Z-nonadecadienoic acid	Hershelman D, Kahler KM, Price MJ, et al. Oxygenation reactions catalyzed by the F557V mutant of soybean lipoxygenase-1: Evidence for two orientations of substrate binding. Arch Biochem Biophys. 2019;674:108082. doi:10.1016/j.abb.2019.108082
panaxydol	Guo Y, Hu M, Ma J, et al. Protective effect of panaxydol against repeated administration of aristolochic acid on renal function and lipid peroxidation products via activating Keap1-Nrf2/ARE pathway in rat kidney. J Biochem Mol Toxicol. 2021;35(1):e22619.

	doi:10.1002/jbt.22619
α-cyperene	Ahn JH, Lee TW, Kim KH, et al. 6-Acetoxy Cyperene, a Patchoulane-type Sesquiterpene Isolated from Cyperus rotundus Rhizomes Induces Caspase-dependent Apoptosis in Human Ovarian Cancer Cells. Phytother Res. 2015;29(9):1330-1338. doi:10.1002/ptr.5385
Hepanal	Wu Y, Zhang F, Yang K, et al. SymMap: an integrative database of traditional Chinese medicine enhanced by symptom mapping. Nucleic Acids Res. 2019;47(D1):D1110-D1117. doi:10.1093/nar/gky1021
quercetin	Shen CY, Jiang JG, Yang L, Wang DW, Zhu W. Anti-ageing active ingredients from herbs and nutraceuticals used in traditional Chinese medicine: pharmacological mechanisms and implications for drug discovery. Br J Pharmacol. 2017;174(11):1395-1425. doi:10.1111/bph.13631
